# Water Treatment Process

**DOI:** 10.3390/membranes12050543

**Published:** 2022-05-23

**Authors:** Szilárd S. Bucs, Nadia Farhat, Luca Fortunato

**Affiliations:** 1 Water Chemistry and Water Technology, Engler-Bunte-Institut, Karlsruhe Institute of Technology, 76131 Karlsruhe, Germany; szilard.bucs@kit.edu; 2 Water Desalination and Reuse Center (WDRC), Biological & Environmental Science & Engineering Division (BESE), King Abdullah University of Science and Technology (KAUST), Thuwal 23955-6900, Saudi Arabia; nadia.farhat@kaust.edu.sa

Water scarcity is the main factor driving the enhancement of available technologies and the development of new technologies. Due to the growing domestic and industrial demand, the use of membrane technologies in water treatment processes for water reuse applications has rapidly increased. Due to their ability to produce high-quality water from different types of water sources, membranes play a strategic role and are able to meet future global challenges in the context of the water–energy nexus. Membrane technologies are widely employed for the production of drinking water (i.e., seawater desalination) as well as the treatment and reuse of domestic and industrial wastewater. However, major challenges and opportunities in this field include improvements in several aspects, such as pre-treatment, membrane fabrication, fouling control, selectivity, cost efficiency, resource recovery and process hybridization. 

This Special Issue, entitled “Water Treatment Process” and presented in the journal *Membranes*, aims to assess the latest achievements in membrane-based water process technologies. This Special Issue covers a variety of topics, including the optimization of membrane fabrication conditions for efficient ion removal performance, the evaluation of pre-treatment effects on fouling in membrane distillation, the impact of microplastics on forward osmosis processes for the reclamation of wastewater, the nutrients recovery from wastewater by the electrochemical precipitation of struvite, and the effect of pre-oxidation on the treatment of river water with the ceramic membrane. A total of five original research articles have been published in this Special Issue, and a summary of each article is reported below. 

Membrane selectivity and permeability play a key role in treatment processes due to the increasing complexity and range of contaminants in water streams. In this context, the application of electrospun nanofibrous membranes (ENMs) has gained attention in water treatment and desalination processes due to their high porosity and high surface-to-volume ratio. However, it must be noted that unraveling the relationship between the fabricating conditions and ENMs morphology is complex. Younes et al. [1] adopted a sophisticated data mining technique called principal component analysis (PCA) to better understand the role of the fabricating conditions of ENMs. The authors evaluated the influence of membrane fabrication conditions, characteristics, and process conditions in the fabrication of different ENMs. The membranes were classified into two main categories: single-layered and multiple-layered. For both cases, the authors did not separate the variables of fabrication conditions from the membrane characterization variables. The presented results show that the ENMs characteristics depend on their fabrication conditions and chemical compositions. In the case of single-layered ENMs, the PCA highlighted the relationship between the textural properties of membranes, and the PVDF layer and acetone added to the support layer. In the case of multiple-layered ENMs, the results reveal the central effect of PVDF moieties, affecting pore size, water contact angle, porosity (membrane characteristics), high voltage, and liquid diameter (membrane fabrication). The results of this study demonstrated the key role of ENMs fabrication conditions on membrane performance. 

Membrane separation processes were proposed in several different treatment schemes for the reuse of water and valuable by-products from wastewater. In this context, Wang et al. [2] evaluated the feasibility of a forward osmosis system to reclaim water for irrigation purposes from wastewater containing nano- and microplastic particles. They found that in the presence of nano- (0.1 μm) and micro (1 μm)-plastic particles, the hydraulic resistance of the fouling layer decreased compared with the control experiments when no particles were present. This was explained by the impact of the particles on the structure of the fouling layer. Moreover, the authors demonstrated that the proposed fertilizer-driven forward osmosis system was suitable for water reclamation from wastewater for irrigation purposes.

Chen et al. [3] evaluated the potential of struvite electrochemical precipitation from swine wastewater; the initial pH, current density and organic substance concentration in the process were evaluated. They found that, under optimal conditions (pH of 7 and current density of 4 mA·cm^−2^), and in the absence of organic substances, the removal efficiency and purity of the precipitate were as high as 96%. However, in the presence of organic substances, the electrochemical precipitation of struvite was significantly inhibited. Nevertheless, the authors showed that precipitation inhibition could be mitigated by increasing the initial pH from 7 to 8 for an extended period. This study showed the potential to electrochemically recover nutrients from anaerobically treated swine wastewater.

In membrane separation processes for water treatment, it is of utmost importance to control membrane fouling in water treatment processes as it deteriorates system performance and reduces the quantity and quality of water. Therefore, the pre-treatment of water before entering membrane systems was addressed in many studies to prolong and maintain membrane performance. Santos et al. [4] evaluated the employment of pre-treatment before membrane distillation using experiments coupled with mathematical modeling. Their results showed that particulate and precipitated material deposition occurred in all tests. However, the fouling mechanism and dynamics were influenced by the type of pre-treatment used (filtration or filtration associated with a pH adjustment). By employing filtration and adjusting the solution pH, a fouling formation could be mitigated in membrane distillation when processing concentrated saline effluent. Based on their results, there is no general strategy to reduce fouling, and it seems imperative to conduct experiments to determine the fouling mechanism of specific effluents. The authors conclude by discussing the technical feasibility of membrane distillation when pre-treatments are employed to mitigate fouling. 

Xia et al. [5] conducted their study to evaluate the efficiency of a pre-oxidation-coagulation flat ceramic membrane filtration process using different oxidant types and dosages in water treatment and a membrane fouling control. First, three different oxidants, including potassium permanganate (KMnO_4)_, sodium hypochlorite (NaClO) and ozone (O_3_), were used as pre-oxidants to study the effects of a pre-oxidation process on the fouling rate of a ceramic membrane filtration of water from the Yangtze River. The pre-oxidation treatment significantly lowered membrane fouling by natural water during continuous filtration, and the degree of mitigation was related to the type and concentration of the oxidants. Then, the different oxidants were used in a coagulation/ceramic membrane combination process. Comparing the three oxidants, the KMnO_4_ pre-oxidation could not maintain the long-term operation of the coagulation/ceramic membrane system. Under suitable concentration conditions, the effect of the NaClO pre-oxidation combined with the coagulation/ceramic membrane system was better than that of the O_3_ system. Oxidants combined with the coagulation/ceramic membrane system were superior to oxidants combined with the ceramic membrane system in terms of the membrane fouling control and water purification effect. The impact of the oxidant on the filtration system was linked to its oxidizing ability and other characteristics. NaClO and O_3_ were more efficient than KMnO_4_. NaClO was better at removing DOC, while O_3_ was more conducive to the removal of UV_254_.

In summary, the novel findings from these studies confirmed the central role of membrane systems in the water treatment processes of different water sources. The use of membrane systems offers a great advantage in terms of flexibility, where the type of membrane and process can be chosen based on the application or the desired water quality. Moreover, the presented results advance our understanding of membrane processes and are promising for future development. The research gaps identified in this Special Issue highlight the challenges and opportunities of membrane-based water process technologies.
